# Touch increases autonomic coupling between romantic partners

**DOI:** 10.3389/fnbeh.2014.00095

**Published:** 2014-03-27

**Authors:** Jonas Chatel-Goldman, Marco Congedo, Christian Jutten, Jean-Luc Schwartz

**Affiliations:** Grenoble Images Parole Signal Automatique (Gipsa-lab), Institut Polytechnique de Grenoble, CNRS, UMR5216, Université Joseph Fourier, Université Pierre-Mendès-France, Université Stendhal - GrenobleFrance

**Keywords:** affective touch, empathy, social interaction, interpersonal coupling, electrodermal activity, physiological coupling

## Abstract

Interpersonal touch is of paramount importance in human social bonding and close relationships, allowing a unique channel for affect communication. So far the effect of touch on human physiology has been studied at an individual level. The present study aims at extending the study of affective touch from isolated individuals to truly interacting dyads. We have designed an ecological paradigm where romantic partners interact only via touch and we manipulate their empathic states. Simultaneously, we collected their autonomic activity (skin conductance, pulse, respiration). Fourteen couples participated to the experiment. We found that interpersonal touch increased coupling of electrodermal activity between the interacting partners, regardless the intensity and valence of the emotion felt. In addition, physical touch induced strong and reliable changes in physiological states within individuals. These results support an instrumental role of interpersonal touch for affective support in close relationships. Furthermore, they suggest that touch alone allows the emergence of a somatovisceral resonance between interacting individuals, which in turn is likely to form the prerequisites for emotional contagion and empathy.

## Introduction

A warm touch can convey more than a thousand words. In this era of exacerbate virtual communication we are faced with a shortage of tactile stimulation, which has been referred to as the “touch hunger” (Field, 2001). The critical role of interpersonal touch for human development, health, communication of affect, and intense social bonding is currently attracting an increasing interest in the emerging field of social neuroscience (Morrison et al., 2010).

To date most neuroscience research on affective touch has focused on investigation at the individual level. As it has been argued by Schilbach (2010), considering isolated individuals cannot account for all mechanisms subtending online social interaction. By contrast, bringing natural and reciprocal interaction into experimental paradigms—in addition to simultaneous data collection from multiple subjects—allows the exploration of interpersonal dynamics and coupling (Chatel-Goldman et al., 2013). The latter phenomenon—also referred to as “interactive alignment,” “resonance,” “linkage,” “synchronization,” “neurobehavioral coordination,” etc.—deals with the association among the activities of interacting individuals. To the best of our knowledge, these non-local mechanisms have never been investigated during interpersonal touch.

There is increasing evidence that during social interaction interpersonal coupling not only occurs at behavioral or even brain level (see e.g., Oullier et al., 2008; Krueger and Michael, 2012), but also at a more general physiological level. This may be especially true when intense affect is experienced by people linked by strong emotional bonds. Such circumstances can lead to high empathy or emotional contagion between individuals (see Box 1 for operational definitions). These social processes have been shown to rely at least in part on our automatic tendency to mimic the expressions of others (Decety and Jackson, 2006). Ethological observation has supported the view that covert mimicry of a target triggers in the observer the autonomic response associated with that bodily state (Preston and de Waal, 2002). Alignment in the physiological response of two people was first demonstrated more than 50 years ago during psychotherapy (DiMascio et al., 1957; Malmo et al., 1957). Based on a priming study on romantic couples (Levenson and Gottman, 1983), Levenson and Ruef (1992) found increased accuracy in rating negative emotional states when both individuals exhibited higher synchrony in their skin conductance and time of pulse transmission from heart to the fingers. In other words, the closer the physiological states of two individuals, the more accurate they are at perceiving the feeling of each other. More recently, in a field observation carried out during a collective fire-walking ritual, Konvalinka et al. (2011) identified synchrony over time of heart rate dynamics between active participants with their related observers, but not with their unrelated observers. In another ecological study, Müller and Lindenberger (2011) found oscillatory couplings of cardiac and respiratory activity among singers and conductor engaged in choir singing. Taken together, these works provide evidence for a shared physiological substrate of empathy. Although interpersonal touch plays a singular role for emotional support and communication in many respects, the extent to which it relates with autonomic coupling is still completely unknown.

Box 1 **Operational definitions.**

- **Emotion contagion/vicarious emotion/emotion transfer:** Low-level, implicit, fast, and automatic affect sharing with no self-other distinction (De Vignemont and Singer, 2006).
- **Empathy:** Capacity to understand and respond to the unique affective experiences of another person (Decety and Jackson, 2006).
- **Splitting with emotions:** Defensive blockage of reflexive affective processes involved in emotion contagion and empathy (Favre et al., 2005).
- **Proximate:** Responses of the individual (and his organs) to immediate factors of the environment (Preston and de Waal, 2002).

Touch is the first sense developed in the womb and as such it can support parent-infant interaction even before birth (Gallace and Spence, 2010). This has been instantiated by the development of pre-post natal approach of haptonomic care, which allows the parents to form affective and concrete ties with their child very early in the pregnancy (Dolto-Tolitch, 1997). In adults, interpersonal tactile stimulation may foster intense bond and strengthen romantic relationships (Gallace and Spence, 2010). Affective dimension of touch was unveiled recently with the key discovery of human C-tactile (CT) afferent, which show a preference for tactile information with socio-affective relevance (Löken et al., 2009; Morrison et al., 2010). Existence of these “labeled-line pathways” (Adolphs, 2010) transmitting affective properties of social touch may explain in part the fact that pleasant touch intensifies the emotional experience conveyed by other modalities (Knapp et al., 2012), conveys hedonic aspects of emotions and is able to communicate distinct emotions (Hertenstein et al., 2006). Interestingly, healthy individuals with high autistic traits exhibit disruptions in the neural system associated with affective touch processing (Voos et al., 2012). Finally, it was demonstrated that the mere observation of touch in another human activates somatosensory cortex in healthy subjects (Blakemore et al., 2005). Recent works (Banissy and Ward, 2007; Keysers and Gazzola, 2007) favor the existence of a tactile mirror system contributing to the somatosensory dimension of simulation processes involved during empathy.

In summary, the emergence of a common physiological substrate may constitute the necessary basis for shared affective representations (and their efficient communication) during social interaction. In this article we study social touch to provide experimental evidence supporting this idea. We believe that interpersonal touch is essential in promoting the formation of an intersubjective state of physiological synchrony. Thus, we hypothesized that during empathy touch would increase coupling of autonomic activity between romantic partners and such attunements would be correlated with social traits of personality. In addition, we expected interpersonal touch to intensify emotional display and feeling in participants, thereby inducing major changes in various measures of autonomic activity. In order to test these hypotheses, we designed an ecological paradigm allowing mutual interaction by touch of romantic partners while simultaneously collecting three different measures of autonomic activity: respiration, heart activity through pulse and electrodermal activity. Social traits of personality were assessed by means of an empathy questionnaire. During the experiment, emotion was induced in a naturalistic fashion by auto-biographic recall of intense affective events.

## Methods

### Participants and ethics statement

Dyads were chosen as romantic couples for the following reasons: (1) it has been suggested that exchange of affective information is stronger between closely attached individuals (De Vignemont and Singer, 2006) and (2) affective touch plays a fundamental role as a means of interpersonal communication in romantic relationships (Gallace and Spence, 2010). 14 different-sex romantic couples (mean age of women 25.4 ± 3.5 years, mean age of men 26.1 ± 3.7 years) participated in the study. They had been engaged in a romantic relation for at least 6 months at the time of the scanning (mean 2.9 years, range 6 months to 5 years). All participants provided written informed consent after receiving a detailed explanation of the experimental procedure, and received a compensation for their participation to the study. Participants were excluded if they had a history of neurological disorder such as seizure, stroke, head injury or epilepsy, or history of psychotropic substance abuse except nicotine and caffeine. Furthermore, they were required to be free of psychotropic substances other than caffeine or nicotine before the experiment. The local ethics committee “Comité d'Ethique pour les Recherches Non Interventionnelles” (University of Grenoble) approved all experimental procedures for this study.

### System used for dual electrophysiological data recording

We designed a system that allowed for simultaneous collection of electrophysiological data in two individuals. All hardware devices used in this study have been produced by “g.tec” (Graz, Austria). For each subject we recorded:

- *Respiration movement*, using an elastic respiratory belt (g.RESP piezoelectric sensor). The belt was placed around participant's chest just below the axilla for transduction of rib cage circumference.
- *Pulse*, using a photoplethysmographic sensor (g.PULSE). The light sensor was attached to the left index finger, facing volar surface on the distal phalange.
- *Electrodermal activity*, using a galvanic skin conductance sensor (g.GSR). Two electrodes were placed on left middle and ring fingers, facing volar surface on the distal phalange.
- *EEG activity*, using a 24 electrodes EEG setup. We do not give more details on EEG acquisition as these data are not analyzed in the present study.

All these sensors were connected to four amplifiers (g.USBamps) sharing the same clock, thus ensuring the exact synchronization of all samples for all sensors. Signals were analog filtered between 0.01 and 100 Hz with a 50 Hz Notch rejection filter, amplified and digitalized at 512 Hz with a 24-bit resolution. Additionally, signals from all sensors were acquired using different grounds and references for each subject to avoid artifact contamination across subjects and/or modalities. Finally, subjects wore an antistatic wrist strap connected to identical ground (earth) to reduce environmental electromagnetic contamination in biosignal recordings.

### Experimental procedure

At the beginning of the experiment participants were briefed on the different tasks they had to perform. They were instructed to select four personal life events—preferably experienced together—associated with an intense emotional experience. Among these four life events, the partners had to choose together two events associated with a positive emotion and two events associated with a negative emotion. It was made clear that these information was private and not recorded. Particular care was also taken to ensure that participants took as much time as needed to agree on their recall. The experimenter leaved the room during the discussion of the couple.

**Figure 1:**
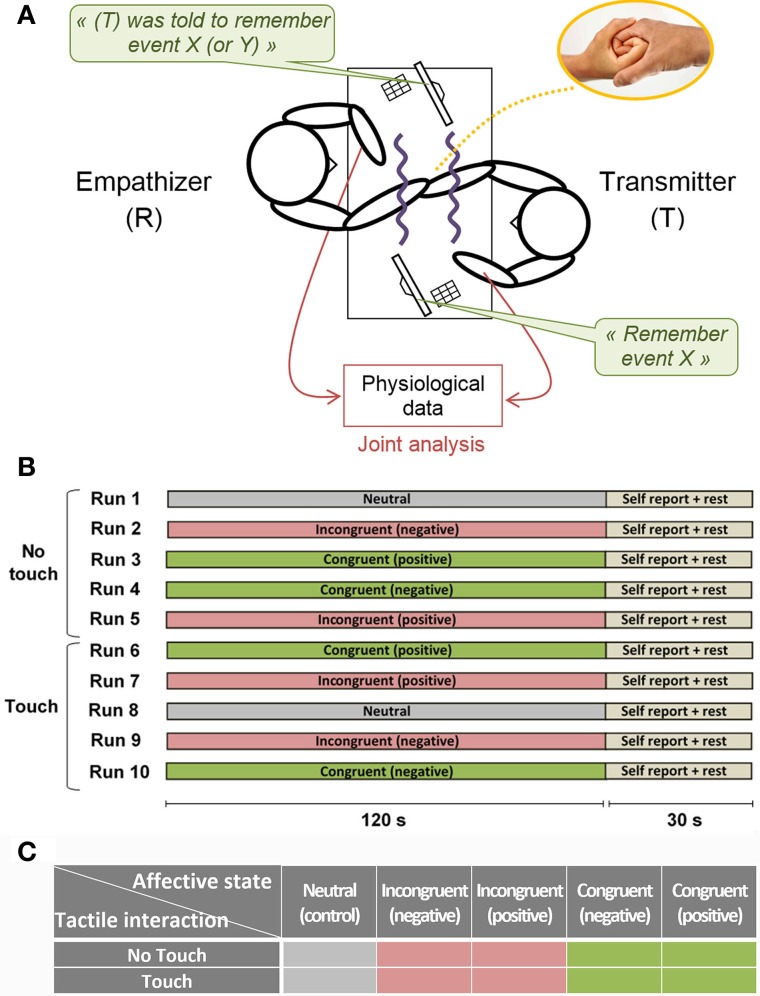
**Experimental setup. (A)** Schematic view of the experimental apparatus. Visual contact between subjects was completely prevented using two opaque partitions. Screens and input devices allowed giving unique instructions to each subject and collecting subjective feedbacks throughout the experiment **(B)** Experimental design. During the five first runs any information exchange is prevented between subjects. During the five last runs, partners can freely touch each other's hand and forearm in a concealed space between opaque partitions. Within Touch or No Touch blocks the run order is randomized. At the end of each run participants reported subjective feedbacks about emotion intensity and perceived coupling with their partner. **(C)** Factorial design.

Once they had selected life events participants were installed in the experimental room with electrophysiological measurement devices. During the experiment visual contact between subjects was completely prevented using two opaque partitions. Screens and input devices were employed to give unique instructions to each subject and to collect subjective feedbacks throughout the experiment (Figure 1). We evaluated the empathy level of each subject through an empathy test (see Behavioral data analysis) and implicitly assigned the role of Empathizer (E) to the subject with the highest score and the role of Transmitter (T) to the subject with the lowest score.

The experiment consisted of 10 runs lasting 2 min each (Figure 1). During the first five runs any information exchange was prevented between subjects (No Touch condition). During the last five runs, romantic partners could freely touch each other's hand and up to half of the forearm in a concealed space between opaque partitions (Touch condition). At the start of each run participants received instructions on their personal screen. Participant (T) was asked to recall one event among the four events selected previously, either positive or negative. Participant (E) was notified about the chosen event and was instructed to be as empathic as possible, not focusing on the recalled event itself, but trying to share his/her partner's emotional state. In half or the runs, the Empathizer priming was congruent with instructions given to the Transmitter (“Congruent” condition), that is, we instructed (T) to remember event X, and we informed (E) that (T) was asked to remember event X. However, in another half of the runs, we deceived the Empathizer by providing him with incongruent priming (“Incongruent” condition), that is, we instructed (T) to remember event X, and we informed (E) that (T) was asked to remember event Y. Using such congruent or incongruent priming of the Empathizer subject allowed us to implicitly manipulate empathy and to avoid explicit instruction to communicate and/or decode the emotions. We also introduced two neutral runs to control for the emotional arousal of the participants (“Neutral” condition). In this condition (T) was instructed to recall a neutral event such as walking on a familiar street, while (E) was instructed to make no particular effort in sharing the feeling of the partner. The order of the runs within Touch and No Touch blocks was randomized. At the end of each run participants reported subjective feedbacks (see Behavioral data analysis).

### Behavioral data analysis

#### Testing for empathy

The empathy of participants as a personality trait was evaluated using the CEC (Cut-Empathy-Contagion) scale questionnaire (Favre et al., 2005). We choose CEC scale instead of other tests such as BEES (Mehrabian, 1997) or EQT (Lawrence et al., 2004), because:

1. It is the only scale that distinguishes between three components of empathy, emotional contagion, and splitting with emotions (Box 1).
2. It is characterized by high validity, good test-retest reliability and internal consistency (Favre et al., 2009).
3. It was initially designed in French and not translated from another language.
4. It is freely available.

CEC comprises 36 items, i.e., 12 items for each dimension, associated to a five-point Likert scale. In each dimension items are summed after being transposed in the range (0–1) to obtain the final scores. For choice of Empathizer we derived a global empathy score with the following weighting: global = 2 × (empathy) + 1 × (contagion) − 1 × (emotional cut).

#### Online subjective feedbacks

At the end of each run participants reported subjective feedbacks on (1) how intense was the emotion they felt during the run, and (2) how much they perceived they were on “the same wavelength” with their partner. This was accomplished by filling a questionnaire with two items in a 1–10 monopolar rating scale. This online subjective feedback provides us with a unique window on the introspective experience of the participants.

### Physiological data analysis

#### Measure of pulse rate variability

When considering heart beat variations, the gold standard technique consists in estimating Heart Rate Variability (HRV) from the varying length of cardiac cycles in the electrocardiographic signal. It is well established that HRV can be a reliable parameter indicating general psychic and somatic fitness (Schäfer and Vagedes, 2013) and that it plays an important role in emotion regulation (Lane et al., 2009) as well as in social communication (Quintana et al., 2012). In the present study, instead of cardiac cycle interval we use pulse cycle interval as they are determined from photoplethysmographic signal. This option is often referred to as Pulse Rate Variability (PRV). It has been demonstrated that PRV is an accurate estimator of HRV when applied to healthy subjects at rest (Schäfer and Vagedes, 2013), a requirement that is satisfied in our study.

PRV is simply derived from pulse cycle intervals. However, the estimation of such intervals is not straightforward and may require advanced processing steps due to the smooth nature of pulse waveform (Hayano et al., 2005). To obtain PRV from pulse signal we designed a simple and effective method. First we decimate pulse signal from 512 to 32 Hz and remove its mean value. Then we estimate the position of pulse fiducial points by detecting the maxima of its second derivative. Pulse cycle intervals are obtained taking the time differences in successive fiducial points. Finally these intervals are low-pass filtered at 1 Hz and spline-interpolated to get a PRV signal with as many time samples as original data.

From PRV we can get a number of common variables in the time domain and in the frequency domain according to Task Force definitions (Task Force, 1996). In time domain we measure standard deviation (PRV-SD), which gives an index of global power. In frequency domain we compute three variables: mean power in low frequency range 0.04–0.15 Hz (PRV-LF), mean power in high frequency range 0.15–0.4 Hz (PRV-HF), and their ratio (PRV-LF/HF).

#### Measure of respiration volume per time

Respiratory fluctuations were characterized as the Respiration Volume per Time (RVT) as proposed by Birn et al. (2006). Specifically, we estimated the amount of air inspired as the difference between the maximum and minimum belt position at the peaks of inspiration and expiration, respectively. This difference was further divided by the duration of the respiration, i.e., the time between the peaks of inspiration and expiration, resulting in the RVT. Finally, the RVT time series was spline-interpolated to match the chosen sampling rate.

#### Measure of electrodermal activity

Two main components, tonic and phasic EDA, need to be evaluated separately (Boucsein et al., 2012). Tonic-level EDA relates to the low frequency and background components of the signal. Phasic-level EDA relates to the fast components of EDA. The Skin Conductance Responses (SCR) may be elicited by distinct stimuli—in which case they are referred to as Event-Related SCR (ER-SCR) —or may occur in the absence of obvious external stimuli—in which case they are referred to as Non-Specific SCR (NS-SCR).

Because we hypothesize that short-lasting changes in EDA are better suited for the investigation of dynamical coupling with others, in this study we are mainly interested in the analysis of phasic EDA. Accordingly, signal from galvanic sensor is high-passed at frequency cut 0.02 Hz to ensure that SCR are not affected by slow waves and DC components. Finally, EDA is normalized to have maximal value equal to one in order to facilitate intersubject averaging and comparison.

### Analysis procedure

Two-Way repeated measures analyses of variance (ANOVA) were conducted to compare the effect of *Touch* (two levels: “Touch” and “No Touch”), *Empathy State* (five levels: “Neutral,” “Incongruent Positive,” “Incongruent Negative,” “Congruent Positive,” “Congruent Negative”—see Figure 1) and their interaction on behavioral and physiological variables. We used Greenhouse-Geisser sphericity correction for repeated measure ANOVA. *Post-hoc* comparisons were conducted using nonparametric approximated permutation tests (random permutations) to contrast two sample means with paired observations (Edgington and Onghena, 2007) and adjusted with Bonferroni procedure.

## Results

In this study we aim at investigating the effect of affective touch on the dynamical coupling of physiological activities between romantic partners. With this goal in mind, we also extend previous research quantifying the somatic changes induced by touch during social interaction. Finally, we examine how these effects are modulated by empathy as a personality trait, both at individual and inter-individual level.

### Online questionnaires

**Figure 2:**
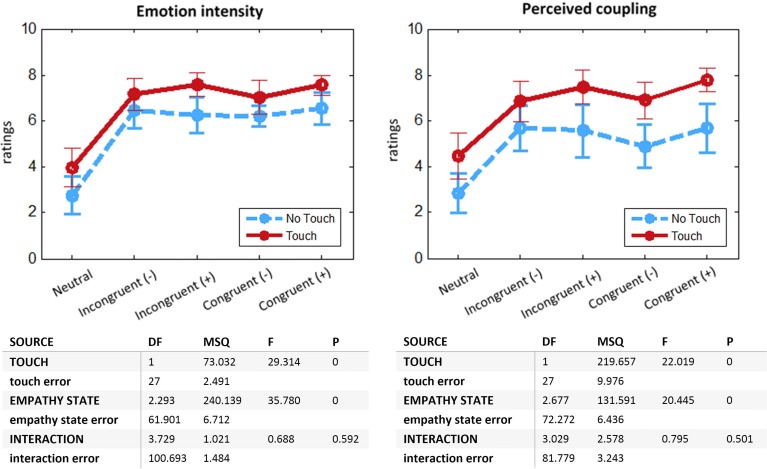
**Online self-report ratings: means with 95% CIs and ANOVA tables**. **Left:** Emotion intensity felt during previous run. **Right:** Perceived coupling with romantic partner during previous run. Two-Way ANOVA with repeated measures, departure from sphericity corrected with Greenhouse-Geisser method. *N* = 28.

We compared the effect of *Touch* and *Empathy State* on self-reports of emotion intensity and perceived coupling with romantic partner. We found similar results for both dependent variables (Figure 2). Participants felt more intense emotions [*F*_(1, 27)_ = 29.31, *p* < 0.0001] and perceived coupling [*F*_(1, 27)_ = 22.19, *p* < 0.0001] for the Touch as compared to the No Touch condition. There was also a main effect of the factor *Empathy State* [emotion intensity: *F*_(2.3, 61.9)_ = 35.78, *p* < 0.0001; perceived coupling: *F*_(2.7, 72.3)_ = 20.45, *p* < 0.0001], but no significant interaction. *Post-hoc* comparisons showed a significant effect for *Emotion* (“Incongruent Positive,” “Incongruent Negative,” “Congruent Positive,” “Congruent Negative”) vs. the Neutral condition (*p* < 0.001), indicating that subjects managed to immerse into an affective state. No significant effect was found for Congruent vs. Incongruent conditions, which implies that in general participants did not notice the manipulation of the Empathizer subject. This result is corroborated by the debriefing after the experiment: 12 out of 14 couples did not mention any perceived incongruence. More importantly, it shows that in the context of our study touch did not permit to communicate emotions with distinct valences. On the other hand, the strong effect of the *Touch* factor on emotion intensity supports the role of touch as an intensifier of affective processing.

### Physiological data analysis at single-subject level

#### Pulse rate variability

**Figure 3:**
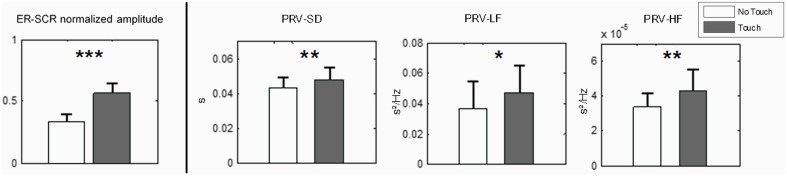
**Physiological measures at single-subject level (means with 95% CIs). Left:** Electrodermal Activity, normalized amplitude of event-related skin conductance response at run's beginning. **Right:** variables of Pulse Rate Variability SD, LF, and HF. *N* = 28. Paired nonparametric permutation test, ^***^ indicates *p* < 0.001, ^**^ indicates *p* < 0.01 and ^*^ indicates *p* < 0.05.

For each PRV variable we conducted a Two-Way ANOVA as described in Analysis procedure. Results were similar for all variables with a main effect of *Touch* [PRV-SD: *F*_(1, 27)_ = 8.10, *p* < 0.01; PRV-LF: *F*_(1, 27)_ = 7.72, *p* < 0.05; PRV-HF: *F*_(1, 27)_ = 7.75, *p* < 0.01], no significant main effect of *Empathy State* and no significant interaction between *Touch* and *Empathy State*. Paired *post-hoc* comparisons for *Touch* are reported in Figure 3. During Touch condition we observed a significant increase of PRV-SD (*p* < 0.01), PRV-LF (*p* < 0.05), and PRV-HF (*p* < 0.01) as compared to No Touch condition.

#### Respiration volume per time

The same ANOVA was performed for RVT. No significant main effect nor significant interaction was found.

#### Electrodermal activity

**Figure 4:**
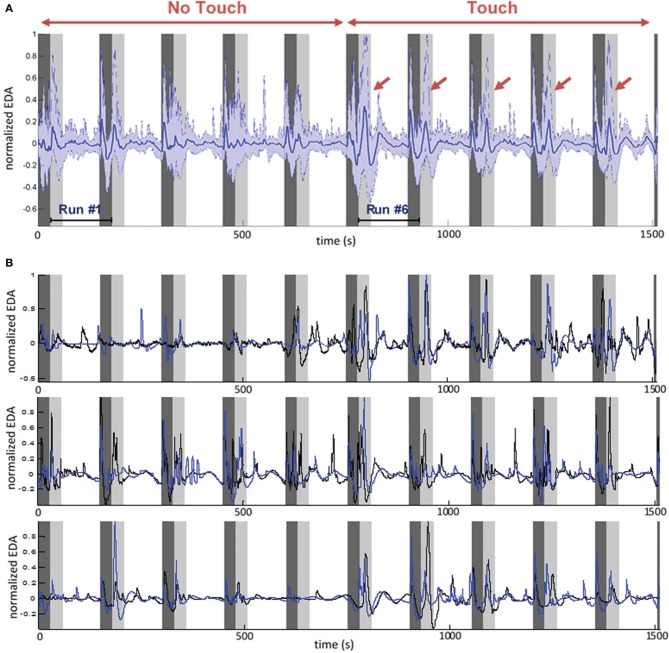
**Time course of electrodermal activity (EDA) during the entire experiment**. The sequence of 10 runs is shown. Dark gray area: rest periods. Light gray area: first 30 s of each run. Run 1–5: No Touch block. Run 6–10: Touch block. **(A)** EDA averaged for all participants (*N* = 28). Dark blue: mean value. Light blue: 5 and 95th percentiles. Red arrows show touch-related ER-SCR. Note that run sequence may be different for each couple, i.e., different conditions are mixed in this average, but order of Touch/No Touch blocks is always the same. **(B)** Example of EDA individual time courses for three different couples. It is visible here that Touch increases individual physiological dynamics and intensifies patterns of inter-subject synchronization.

Measure of EDA for all participants reveals a systematic pattern in time during the experiment (Figure 4). Taking each run separately we can distinguish two main SCR. A first SCR peak appears after the end of each run, at the beginning of rest period (Figure 4, dark gray band). It should be noted here that all runs terminated with a soft ring bell indicating that subjects could stop their emotional recall and begin filling the online questionnaires. Therefore, the first SCR peak is presumably ER and marks an attention shift, with the subject switching from a focused and introspective state of mind to a relaxed and extrospective state of mind.

A second, stronger, SCR peak appears at the beginning of the runs. While it tends to attenuate along the five first runs (a No Touch condition), we observe a strong and reliable response for the last five runs (a Touch condition). This is again an ER-SCR peak that, arguably, happens due to a steep increase of arousal. For objective assessment we measured its amplitude by simply taking the difference between maximum EDA within the 20 first seconds and EDA baseline value at the beginning of each run. The Two-Way repeated measure ANOVA revealed a main effect for *Touch* [*F*_(1, 27)_ = 19.72, *p* < 0.0001]. There was no significant main effect for *Empathy State* and no significant interaction. *Post-hoc* permutation tests for *Touch* (*p* < 0.001) are presented in Figure 3, left.

This second ER-SCR peak appears to gather contributions from at least two components. A first component is certainly exogenous: it is a response to tactile stimulation when touch begins. This strong component contributes the most to the ER-SCR, as shown by its amplitude increase only for touch condition, as well as by the visual inspection of EDA time courses averaged over all participants (Figure 4, light gray bands). A second component, present in both Touch and No Touch conditions, may be endogenous, arising when subjects begin to immerse into an affective state. This component has a weaker contribution, as indicated by non-significant contrast of Emotion vs. Neutral conditions. The fact that it attenuates along the five first runs may be due to the habituation of subjects to autobiographic recall. These results indicate a strong and reliable response on EDA at touch stimulation onset.

Finally, the electrodermal activity in the white band of Figure 4 appears to be composed of a mixing of slow waves and non-specific responses (NS-SCR). Interestingly, from 30 to 120 s of each run the EDA exhibits a high similarity between partners, especially in the Touch conditions. In the ensuing part of this section we will focus on this time period with the aim to quantify such patterns of intersubject physiological synchronization.

### Physiological data analysis at couple level

We quantify linear relationships between the physiological signals of romantic partners by computing their cross-correlations. For PRV and RVT we process cross-correlation taking the entire run length, i.e., keeping time interval 0–120 s. For electrodermal activity we remove the first 30 s to avoid that high amplitude touch-related ER-SCR drives most of the correlation. We compute cross-correlation for each run, apply a Fisher z-transform and take the average for interval (−1, 1 s) around maximum value. This way we obtain a robust estimation of maximum z-scores for each condition. Finally, the scores for EDA are transformed (cubic root) to satisfy the ANOVA assumption of normally distributed data.

**Figure 5:**
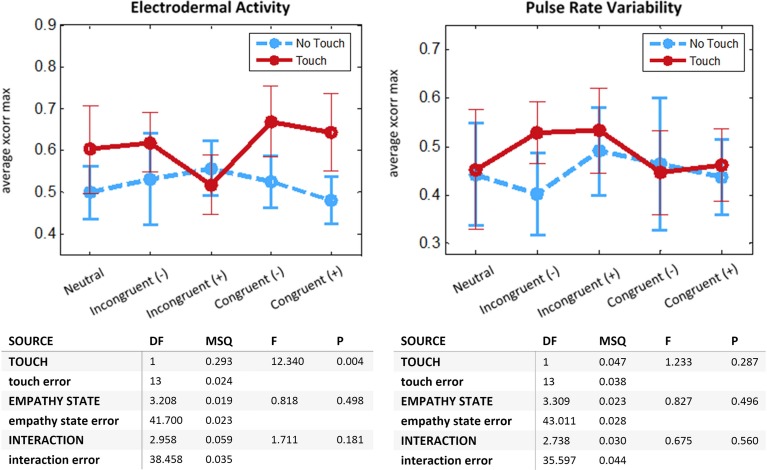
**Physiological coupling between romantic partners**. *N* = 14 couples. For each run we compute the average cross-correlation after Fisher z-transform for interval (−1, 1 s) around maximum value. **Top:** Means with 95% CIs. **Bottom:** Two-Way ANOVA with repeated measures, departure from sphericity corrected with Greenhouse-Geisser method. **Left:** Electrodermal Activity. **Right:** Pulse Rate Variability.

Average intersubject cross-correlations for EDA and PRV are given in Figure 5. The 2-way repeated-measures ANOVA on EDA displayed a main effect for *Touch* [*F*_(1, 13)_ = 12.34, *p* < 0.005]. There was no significant effect of *Empathy State* and no significant interaction. This result demonstrates that interpersonal touch intensifies coupling of electrodermal activity across romantic partners. For PRV and RVT no significant main effect nor significant interaction was found.

**Figure 6:**
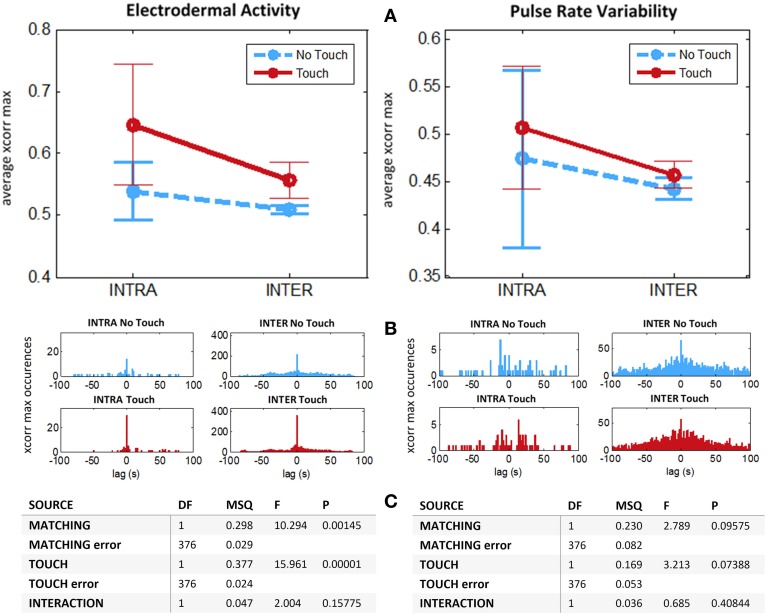
**Average intersubject maximum cross-correlations for experimental (INTRA) and surrogate (INTER) data**. *N* = 14 couples. For each run we compute the average cross-correlation after z-transform for interval (−1, 1 s) around maximum value. Left: Electrodermal Activity. Right: Pulse Rate Variability. **(A)** Means with 95% CIs. **(B)** Histograms of time lags for maximum cross-correlations. **(C)** ANOVA table for mixed design, *Touch*: repeated measures factor (Touch vs. No Touch), *Matching*: independent measures factor. (Intra vs. Inter).

Does higher cross-correlation during tactile stimulation relate to similarities in our physiological response to touch, or is it an emergent property specific to the dynamical coupling? We introduce surrogate data in order to disentangle what is common to all subjects from what is specific to their interaction. This is realized by measuring cross-correlation between subjects from different couples (condition “Inter”). This is the appropriate condition for controlling whether the increase of physiological coupling is due to subject's contingent tactile interaction, or whether it is due to a phase-locked stereotypical response to touch. For instance, it has been employed as a control condition in several hyperscanning studies (e.g., Astolfi et al., 2010; Jiang et al., 2012; Yun et al., 2012). For each run, considering *Ns* = 28 participants we process all *Ns* (*Ns* − 2)/2 = 364 different cross-correlations “Inter.” Finally, from these experimental and surrogate data we perform a mixed 2 × 2 ANOVA with *Touch* as repeated measures factor (two levels: “Touch,” “No Touch”) and *Matching* (two levels: “Intra,” “Inter”) as independent measures factor. Such analysis (Figure 6) for EDA reveals significant main effect for *Touch* [*F*_(1, 376)_ = 15.96, *p* < 0.00001] and for *Matching* [*F*_(1, 376)_ = 10.29, *p* < 0.002] on interpersonal coupling. There was no significant interaction. For PRV we found a marginally significant effects for *Touch* [*F*_(1, 376)_ = 3.21, *p* = 0.074] and for *Matching* conditions [*F*_(1, 376)_ = 2.79, *p* = 0.095] and no significant interaction. We note that data variance is very high. This may be due to the fact that the coupling measure is drawn from multiple subjects whose response to the emotional task might be very diverse. Considering our results altogether we hypothesize that an effect for Touch on PRV exists, but that our sample size is insufficient to allow its detection.

Results presented here suggest that: (1) touch induces a stereotypical response on all subjects, as implied by increase of inter-couple cross-correlation for Touch vs. No Touch conditions and absence of interaction; (2) beyond these similitudes, during touch EDA displays dynamics that are genuine to the reciprocal interaction of romantic partners, as demonstrated with significant decrease of average maximal cross-correlation when computed across rather than within couples.

### Correlation between empathy scores and physiological measures

**Figure 7:**
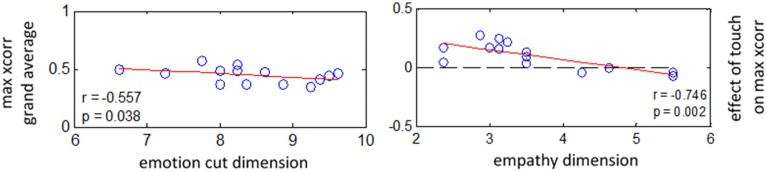
**EDA: correlations between effect of touch on interpersonal coupling and scores at empathy questionnaire**. *N* = 14 couples. **Left:** Spearman correlation between grand average of maximum cross-correlation and score obtained for “splitting with emotions” component. **Right:** Spearman correlation between effect size for touch on intersubject maximum cross-correlation (averaged from lag −1 to +1 s for all conditions) and score obtained for “empathy” component.

Is there a relation between empathy as a personality trait and physiological coupling among romantic partners, and how does it relate with affective touch? We address this by computing correlation between intersubject maximum cross-correlations and the scores obtained from empathy questionnaires (Figure 7). Here we use nonparametric Spearman rank correlation because besides being less sensitive to outliers than Pearson's correlation, it is also sensitive to any form of correlation, not just linear, as long as it is monotonic (Rousselet and Pernet, 2012).

First, we investigated the possible relation between empathy scores and the general level of intersubject cross-correlation, i.e., merging all conditions. For EDA we found a negative correlation between maximum cross-correlation and scores obtained for “splitting with emotions” component [*r*_(26)_ = −0.55, *p* < 0.05]. This shows a trend for partners to be more coupled when they are more empathic, i.e., scoring lower at “splitting with emotions.” Secondly, we inquired whether the effect of touch on physiological coupling is related to the empathy level of participants. Results reveal a negative correlation between the effect size of touch on maximal intersubject cross-correlation and scores obtained for “empathy” dimension [*r*_(26)_ = −0.74, *p* < 0.002]. We did not find any significant relation between empathy scores and cross-correlation for RVT and PRV.

In short, these results indicate a tendency for partners to be more coupled when they are more empathic. In addition, they show that affective touch had a stronger impact on coupling of electrodermal activity with their partners among less empathic subjects.

## Discussion

To the best of our knowledge this study is the first exploration of both interpersonal and reciprocal aspects of human physiology during affective touch and empathy. Analysis at an inter-individual level revealed that during online interaction interpersonal touch increases coupling of electrodermal activity across interactants. We found a similar trend for PRV. Physiological activities displayed dynamics that are genuine to each couple's interaction, as demonstrated by the lower coupling across as compared to within dyads. In addition, analysis at an intra-individual level showed that affective touch induces strong and reliable changes in our physiological states, both for PRV and electrodermal activity.

### Interpersonal physiological coupling during touch is emotion-unspecific

We did not find any significant effect for the manipulation of empathy states on interpersonal physiological coupling. Instead, we found a comparable level of coupling during Neutral (control) condition and conditions when subjects were required to immerse into an emotional state. The validity of the control condition can be verified with subjective reports showing a significant decrease of emotion intensity felt during Neutral condition as compared to the other emotional conditions. This result indicates that touch alone may suffice to enhance interpersonal coupling, regardless the intensity and valence of the emotion felt.

The effects found here for social touch must be considered in light of the context in which touch occurred, i.e., among romantic partners. From an evolutionary viewpoint, dynamic touch in close relationships essentially has a supportive function and it is thought to create a psycho-pharmacological environment in which mutual trust can develop (Dunbar, 2010). It is plausible that interpersonal touch acts more as a facilitator than as a medium for communication of affect. This may be especially true for distant (less intimate) relationships, where individuals usually rely on more explicit channels for emotion display, such as vision and audition. This could explain the fact that the effect of interpersonal touch on physiological coupling is not dependent of whether subjects are actually immersed or not into an emotional state.

### Intimate touch did not allow to communicate distinct emotion

Previous behavioral studies on tactile communication of affect have put forward that touch can communicate distinct emotions (Hertenstein et al., 2006, 2009; Thompson and Hampton, 2011). However, in these works, subjects were not asked to immerse themselves into emotional states, but were rather meant to pose emotional expressions. In their experimental paradigm “encoders” were explicitly prompted to transmit specific stereotypical emotions, and “decoders” were administrated a force-choice response sheet with a list of emotions to retrieve. As a consequence, the exact nature of transmitted information was not clear, i.e., one could not distinguish whether subjects were communicating intentions rather than emotions.

In our study, communication of emotions was assessed implicitly and the task was focused on emotion feeling rather than emotion transmission. On one hand, we found that in this context touch did not permit to communicate distinct emotions, as shown by unnoticed manipulation of Empathizer subjects. This was observed despite the fact that being romantic partners the participants within dyads knew each other very well, and that for incongruent conditions they were submerged into emotions with completely opposed valence. On the other hand we revealed a predominant increase of physiological coupling during touch when measured within rather than across interacting dyads. This provides evidence that touch alone can convey covert information. This haptic social communication, in turn, is sufficient for the dynamical coupling of bodily states between interactants.

Overall, the observations from our study and those from previous literature are not mutually exclusive. Let us advance an integrative interpretation. When people are explicitly asked to encode and decode a tactile message, they can effectively communicate distinct affective intents, as shown in Hertenstein et al. (2006). However, we argue that such overt interpretation of meaningful gestures does not involve emotional processes, but instead rely on more high-level cognitive mentalizing skills in a similar fashion as would necessitate a social game of charades. Here we show that haptic communication is not about stereotypical emotions. We argue rather that physical touch carries covert and partly unconscious information at a very low, implicit level, leaving the door open for our bodies to resonate.

### Which mechanisms allow for interpersonal visceral resonance?

Recent investigations revealed that dynamic social touch is subserved by specific neural pathways, both on peripheral neurophysiology with the CT afferent, and on the cortical level, where these afferent pathways target brain structures associated with affective and homeostatic processing (Morrison et al., 2010). Functionally, CT afferents select a range of velocities likely to have social-affective relevance, preferring speeds within the range of a gentle caress (Löken et al., 2009). Anatomically, CT fibers have more in common with interoceptive and visceral systems than to exteroceptive afferent from sensory discriminative pathways (Morrison et al., 2010). At an individual level, socially relevant tactile stimulations—or even their mere observation (Morrison et al., 2011)—activate these social-affective pathways, which in turn modulate the homeostatic state of the organism through processing of CT information in the insular cortex (Olausson et al., 2010).

Therefore, the physiological state of one individual can be influenced by tactile inputs from another individual. At an inter-individual level, we presume that touch mediates (un)conscious coadaptation of autonomic activities during reciprocal interaction. In our experiment we provided evidence that touch alone conveys sufficient information for allowing a somatovisceral resonance between interacting individuals. We note that for most couples maximal interindividual cross-correlations occurred at lag 0 s, i.e., they were instantaneous, as can be seen on the histogram of their occurrences per time lag (Figure 6). This absence of delay indicates that autonomic synchronies could not be explained by intentional imitation, but rather as an instantaneous, unconscious phenomenon.

In this study, due to the social and dynamic nature of touch between the romantic partners, it is reasonable to assume that CT fibers were recruited when the two partners touched each other. During interpersonal reciprocal touch, these low-level dedicated pathways may play a decisive role for the emergence of a physiological coupling at an interindividual level. According to Olausson et al. (2002), CT fibers can be found on the forearm skin and dorsum of the hand, but not on the glabrous skin of the palm. Using our experimental apparatus (a concealed space between two opaque partitions, see Figure 1) the participants could touch each other's hand and no further than half of their forearm. We did not instructed the subjects to apply specific strokes or movements, but instead they were asked to touch each other freely. Doing so, our aim was to evoke a tactile stimulation similar to their natural touch. The shortcoming of this choice is that in this study we are not able to disentangle the precise type and/or location of interpersonal touch and that we cannot verify the actual recruitment of the CT fibers in this experiment, leaving the question open for further research.

### A supporting hand when empathy lacks

In this experiment we were also interested in exploring how empathy as a personality trait relates with intersubject physiological coupling. Empathy trait scores were evaluated using the CEC scale (Favre et al., 2005), which distinguishes three components: “empathy,” “emotional contagion,” and “splitting with emotions” (Box 1). Favre defines the “splitting with emotions” dimension as an index of our mostly unconscious propensity to distancing from affect when it usually induces suffering and/or loss of control. This dimension implies a partial blockage of processes involved in emotion contagion and empathy. In a validation study on 761 pupils from 8 to 17 years old, it was shown to be more developed in male subjects and to constitute an indirect index of the risks of developing violent behaviors (Favre et al., 2009).

We obtained several correlations between empathy trait scores and interpersonal physiological coupling. First, we found a significant tendency for partners to be more coupled in average when they scored less at the “splitting with emotions” component. This result supports the notion that personal developmental trajectories, in terms of emotion processing, empathy, and affective defensiveness, may be reflected by related abilities to form a visceral connection with others. This observation can be seen as a comforting argument for coupling of autonomic activities to form the prerequisites for empathy. Besides, it is very likely that individuals with pathological social impairments in general present a higher splitting with emotions (albeit this affirmation requires controlled validation). Extrapolating from the tendency we exposed, it is possible that population with pathological social impairments suffers from an impaired ability to create and maintain an interpersonal physiological ground during online social interaction. Likewise, if touch is an essential channel for creating bonds at a physiological level, then disruption in its processing should be associated with disruption in processing and regulation of emotion and in a social context. This conjecture is supported by recent findings that individuals having high autistic traits exhibit disruptions in the neural systems associated with processing of affective touch (Voos et al., 2012). Putative relation between autistic traits and interpersonal physiological coupling during affective touch should be the subject of future investigations.

We observed that touch had a greater effect on physiological coupling among couples who scored lower at empathy items. This may indicate that less empathic romantic partners were also less familiar with the kind of warm and comforting touch that was solicited during experiment, hence they exhibited an increased contrast in their physiological resonance. More importantly, this last result shows that affective touch can bring romantic partners “viscerally” closer, thereby increasing intimacy and providing a remarkable medium for partner support. This gives additional support for the beneficial effects of warm touch on couple's well-being. Whereas touch is part of the oldest treatment modalities, only very recently we have begun to assess its biochemical, physiological, cognitive, and emotional effects in controlled studies (Field, 1998). An increasing body of evidence demonstrates that non-massage warm touch among couples has a beneficial influence on multiple stress-sensitive systems: lower cardiovascular reactivity to stressful life events (Grewen et al., 2003), lower blood pressure (Light et al., 2005), enhanced oxytocin activity, and lower stress hormone levels for both husbands and wives (Holt-Lunstad et al., 2008), decreased symptoms of subclinical depression (Holt-Lunstad et al., 2011). Mere contact pressure of holding a husband's hand has been shown to reduce activation in the neural systems supporting response to an impending threat (Coan et al., 2006). Not to mention that positive effects of warm touch are already widely recognized by therapists in osteopathy, haptonomy, and palliative care. By extending the focus to multiple interacting individuals, in this study we have revealed a physiological coupling across partners during affective touch. This intersubjective mechanism may play an indirect role for stress alleviation during affiliative behaviors by allowing spouses for increased support and reciprocal openness. The findings provided here may help us better understand the protective influence of warm touch in the prevention and care of stress-related diseases in close relationships.

### Conflict of interest statement

The authors declare that the research was conducted in the absence of any commercial or financial relationships that could be construed as a potential conflict of interest.
